# The Involvement of Calpain in CD4^+^ T Helper Cell Bias in Multple Sclerosis

**DOI:** 10.4172/2155-9899.1000153

**Published:** 2013-06-14

**Authors:** Nicole Trager, Jonathan T Butler, Azizul Haque, Swapan K Ray, Craig Beeson, Naren L Banik

**Affiliations:** 1 Department of Neurosciences, Medical University of South Carolina, 96 Jonathan Lucas Street, South Carolina, SC 29425, USA; 2 Department of Microbiology and Immunology, Hollings Cancer Center, Medical University of South Carolina, 173 Ashley Avenue, South Carolina, SC 29425, USA; 3 Vanderbilt Neurosciences, Vanderbilt University, 1211 Medical Center Dr, Nashville, TN 37232, USA; 4 Department of Pathology, Microbiology, and Immunology, University of South Carolina School of Medicine, 6439 Garners Ferry Road, Columbia, SC 29209, USA; 5 Department of Drug Discovery and Biomedical Science, Medical University of South Carolina, 280 Calhoun Street, Charleston, SC 29425, USA

**Keywords:** Calpain, EAE, STAT, Multiple sclerosis, T helper cells, Cytokines

## Abstract

The pathogenesis of multiple sclerosis (MS) is mediated by massive infiltration of myelin-specific T cells into the central nervous system (CNS). Self-reactive CD4^+^ T helper (Th) cells, specifically Th1 and Th17 cells, are hallmarks of active disease in progression, whereas Th2 cells are predominately in remission stages. Calpain has been shown to be upregulated in the CNS of MS patients and inhibition of calpain has been shown previously to decrease disease in experimental autoimmune encephalomyelitis (EAE), an animal model of MS. We investigated calpain involvement in Thcell bias. Here, we show that calpain inhibition in primary myelin basic protein (MBP) Ac1-11-specific T cells and MBP-specific T cell line cultures increase Th2 proliferation, cytokine profile, and transcription and signaling molecules. We also show a relative decrease in Th1 inflammatory factors in these same categories and a relative decrease in Th17 proliferation. These studies provide insight into the various roles that calpain plays in Th cell bias and proliferation and increases our understanding of the role that T cells play in the pathophysiology of EAE and MS. Results also indicate the mechanisms involved by which calpain inhibitor decreases the disease signs of EAE, suggesting that calpain inhibitor can be a possible therapeutic agent for the treatment of EAE and MS.

## Introduction

Multiple sclerosis (MS) is an autoimmune disease of the central nervous system (CNS) for which there is no cure. This is because thus far no agents that initiate inflammation, demyelination, and degeneration in the brain have been identified. Current therapy is focused on immunomodulation. Although its etiology remains unknown, MS is in large part thought to be initiated and mediated by an inflammatory infiltration of self-reactive CD4^+^ T helper (Th) cells into the CNS [1–3]. Inflammatory Th cell bias composed of overactive Th1 and Th17 subtypes that lead to a self-directed inflammatory attack resulting in myelin degeneration and the pathological symptoms that are observed in MS [4–6]. Conversely, anti-inflammatory Th2 cytokines (e.g. IL-4, IL-5, IL-10, and IL-13) and cell type are predominant during disease remission [7–9]. T regulatory (Treg) cells have also been shown to be deficient and dysfunctional in MS [10–12]. T cells differentiate through specific signaling pathways, including various signal transducers and activators of transcription (STAT) molecule subtypes, which transmit the activation response to certain transcription factors for upregulating specific cytokine loci to reinforce the Th cell subtype. There is a cross reactivity in the signaling pathways but taking a simplistic view, Th1 subtype develops from IFN-γ and IL-12 signal through STAT1/T-bet to drive further cytokine expression and subtype reinforcement. Th2 subtype develops from IL-4 and IL-2 signaling through STAT6/GATA3. Th17 subtype develops through TGFβ, IL-1, IL-6, IL-21, and IL-23 signaling through STAT3/RORγt to develop and reinforce the subtype. Finally, the Treg subtype develops through TGFβ and IL-2 signaling though STAT5/Foxp3 to drive further regulatory cytokine secretion [13,14].

While the exact etiology of MS is unknown, both acidic and neutral proteases are thought to play a role in the disease development. One such protease with increased activity in MS is the Ca^2+^-activated neutral protease calpain [15–18]. Calpain is involved with normal cell functioning but also is associated with deleterious events when deregulated as a result of abnormal increases in intracellular free Ca^2+^ concentration. Increased activation of calpain has been shown to be involved in T cell activation and migration as well as in neurodegeneration [19–23]. Calpain degrades myelin as well as axonal proteins and various STAT molecules that are also known substrates of this ubiquitously expressed Ca^2+^-activated protease, calpain [24–27]. Previous studies have shown that activated STAT6 is degraded in T cell hybridoma cells and this degradation can be prevented with calpain inhibitors [28]. STAT6 is known to contribute to the Th2 subtype bias through IL-4 [29,30]. The Th2 subtype also has the ability to down regulate the Th1 and Th17 type cells through cytokine secretion.

Calpain inhibition has been shown to decrease Th1 and Th17 cytokines in peripheral blood mononuclear cells (PBMCs) of the MS patients [31]. In experimental autoimmune encephalomyelitis(EAE), an animal model of MS, disease severity is reduced with calpain inhibitor treatment [31–34].There has also been increased calpain activity in the infiltrating monocytes during acute EAE, which tends to be biased toward the Th1 and Th17 subtypes [27]. The regulation of STAT6 through inhibited degradation and its target transcription factors may be a mechanism through which calpain inhibition provides clinical benefit. Thus, calpain may be used as a probable therapeutic target in the treatment of EAE and MS.

The aim of the current experiments was to investigate the *in vitro* effects of calpain inhibition on Th cell subsets in order to determine if calpain is involved in the promotion of inflammatory Th cells. MBP-specific T cells treated with calpain inhibitor showed an increase in Th2 proliferation, alteration of cytokine profile, and transcription and signaling molecules with a relative decrease in Th1 inflammatory factors in these same categories and a relative decrease in Th17 proliferation. These findings were associated with differences in STAT protein levels and functional location between the two treatment groups. Thus, calpain could be a therapeutic target in MS, which acts partially through the STAT pathway to decrease inflammatory Th cells and promote anti-inflammatory Th cells.

## Materials and Methods

### MBP-specific T cell culture

CD4^+^ Th cells (B10A.E3 or B10A.E2) were originally isolated from peritoneal lymph nodes (LN) of the B10.A-H2a H2-T18^a^/SgSnJmice (Jackson Laboratories, Bar Harbor, ME) following subcutaneous (s.c.) inoculation with MBP Ac1-11 in complete Freund’s adjuvant [35]. The cells were diluted to a single cell culture and stimulated with γ-irradiated (3000 rad) isogenic speloncytes, MBP Ac1-11 and rIL-2 (Invitrogen, Carlsbad, CA). The cells were grown in RPMI 1640 (MediaTech, Herndon, VA) supplemented with 10% fetal calf serum (FCS) (Hyclone, Logan, UT), 10 mM HEPES (Sigma Chemical, St. Louis, MO), 2 mM GlutaMAX (Invitrogen, Carlsbad, CA), and 50 µM β-mercaptoethanol (GIBCO, Grand Island, NY). The cells were restimulated every 7–9 days with a 10-fold excess of γ-irradiated isogenic speloncytes, 50 µM MBP Ac1-11 and rIL-2 and maintained at 6–7×10^5^ cells/ml. Media and rIL-2 were added during the first 72 hours and cells were split as needed to maintain the proper cell concentration. At the time of restimulation the required cells were split off for assays. The MBP-specific cell lines were utilized for the proliferation assay, cytokine profiling, Western blotting, and subcellular localization microscopy. Several treatment groups were tested. Control: no calpeptin and no vehicle, Vehicle: 0.2% DMSO, Unstimulated: no MBP peptide, Stimulated 4K peptide, Doses of 100 nM, 10 nM, 1 nM, 100 pm, 10 pm and 1 pm calpeptin.

### Primary MBP-specific T cell culture

Proliferation assay and cytokine profiling were performed with primary MBP specific cells. B10.PL mice (Jackson Laboratories, Bar Harbor, ME) were injected s.c. with an emulsification of 400 µg of guinea pig MBP in complete Freund’s adjuvant (Difco, Lawrence, KS) at four locations over the back, followed with 200 µg of Pertussis toxin (Sigma) injected intraperitoneally (i.p.) at the time of inoculation and then again 48 hours later. Ten to twelve days later, the draining LNs and the spleen were dissected out and fritted glass slides were used to grind the tissue into a single cell suspension and strained through a 70-µm cell filter (BD Biosciences, San Jose, CA) to remove the larger tissue debris. The LN cells were grown with a 10-fold excess of spleen cells and were stimulated with 50 µM MBP Ac1-11 in the same media as described above.

The various Th subtypes were favored for proliferation with exogenous recombinant cytokines and neutralizing antibodies: for Th1 potentiation, IL-12 (10 ng/ml; R&D Systems, Minneapolis, MN) and anti-IL-4 (2 µg/ml; Santa Cruz Biotechnology, Santa Cruz, CA); for Th2 potentiation, IL-4 (10 ng/ml; Cell Sciences, Canton, MA) and anti-IL-12 (2 µg/ml; R&D Systems); for Th17 potentiation, IL-1β and IL-23 (10 ng/ml; R&D Systems) and anti-IFN-γ and anti-IL-4 (10 µg/ml; R&D Systems); for Treg potentiation, IL-2 (150 U/ml; BD Biosciences) and TGFβ1 (1 ng/ml; R&D Systems) and anti-IFN-γ and anti-IL-4 (10 µg/ml; R&D Systems). The cytokines were chosen not to differentiate naïve Th cells to the respective subtypes but rather to reinforce the proliferation of the differentiated subtypes already present in the culture. Supernatant samples were taken at 24 hours following plating and then culture media were replaced with fresh media and respective cytokines and antibodies. All the treatment groups were compared with the controls to verify cytokine effects. Unstimulated: no MBP peptide, Stimulated 4K peptide, Doses of 100 nM, 10 nM, 1 nM, 100 pM, 10 pM and 1 pM calpeptin.

### Proliferation assay

The proliferation assay was set up on a restimulation day for the MBP-specific T cell cultures or on the day of plating for the primary culture. The assay was performed in 96-well plates (Corning, Wilmington, NC) with 8–9×10^4^ MBP-specific T cells per well and a 6- to 10-fold excess of irradiated (3,000) isogenic speloncytes as antigen presenting cells per well. Cells were activated with either the native MBP Ac1-11 peptide or a MBP Ac1-11 peptide with an alanine substitution at position 4 for a positive stimulation control (a known substitution that transforms the peptide into a strong stimulator of the Tcell receptor) [36]. Various concentrations of calpain inhibitors were added to the wells. No rIL-2 was used in this assay during the stimulation. The primary culture had the appropriate cytokines and antibodies added to the media. After 24 hours, 100 µl of supernatant was removed for cytokine profiling and 2 µCi of ^3^H-thymidine in fresh media was added to each well. After more 24 hours, the cells were lifted with trypsin EDTA (Invitrogen) treatment and harvested with a semi-automated cell harvester (Skatron Instruments, Sterling, VA) onto a filter paper membrane (Skatron, New York, NY). Each well’s filter was added to a scintillation vial (Fisher Scientific, Pittsburgh, PA) with 2 ml of Ecoscint A scintillation fluid (National Diagnostics, Atlanta, GA). A TRI-CARB 2900TR Liquid scintillation analyzer (PerkinElmer, Shelton, CT) was utilized to determine the amount of ^3^H-thymidine as an indicator of DNA replication and proliferation in the cells.

### Cytokine profiling

Cells were grown to desired confluency and either plated in a 96-well plate for proliferation assays or in 6-well plates for large-scale experiments. Cell free supernatant was collected 24 hours after exposure to the specified treatments and frozen at −80°C until the cytokines were assayed. OptIEA Sandwich ELISA (BD Biosciences) was utilized to test for individual cytokine levels in the various exposures following the standard protocol. A matched antibody pair (BD Biosciences) was utilized for IL-17A with a modified ELISA protocol based upon the manufacturer’s kit instructions. The cytokines tested were IL-2, IFN-γ, IL-12p70, IL-4, IL-10, and IL-17A. The cytokine profile of the E3 cells and unbiased primary T cells were further explored using an inflammatory cytokine antibody array (Ray Biotech, Norcross, GA) to more fully characterize the subtype profile. This array is an immunoblot array performed following the kit instructions and detected on a Fluochem FC2 Chemiluminescent CCD detection system (Alpha Innotech, San Leandro, CA). AphaEaseFC (Alpha Innotech) software was used to calculate the densitometry of the various spots in the array and Excel (Microsoft, Redmond, WA) was used to graph the data. We compared unstimulated cells (no MBP peptide) to stimulated cells (with MBP peptide) to cells stimulated with MBP peptide and treated with 100 µM, 10 µM and 1 µM of calpeptin.

### Western blotting

Briefly, the total protein samples were extracted following the lysis with ultrasonic homogenization of control and treated MBP-specific T cells, quantitated spectrophotometrically, denatured in boiling water for 5 min, and loaded onto the SDS-polyacrylamide gradient (4–20%) gels (Bio-Rad Laboratories, Hercules, CA). The proteins were resolved by electrophoresis in Laemmli buffer and then electroblotted to ImmobilonTM-P membranes (Millipore, Bedford, MA). For detection of each specific protein band on the blots, appropriate dilutions (either 1:250 or 1:1000 depending on the supplier) of the primary IgG antibodies were used. STAT1, 4 and 6, T-bet and GATA3 primary antibodies were obtained from Santa Cruz Biotechnology (Santa Cruz, CA). The blots were first incubated with the primary IgG antibody followed by incubation with the appropriate alkaline horseradish peroxidase (HRP)-conjugated secondary IgG antibody (ICN Pharmaceuticals, Aurora, OH). The ChemiGlow (Alpha Innotech, San Leandro, CA) H_2_O_2_ catalyzed substrate solution was added to the blots. The protein bands were then detected with a Fluochem FC2 Chemiluminescent CCD detection system (Alpha Innotech). The images were then processed using AphaEaseFC (Alpha Innotech) to quantify the OD units of the bands. β-actin expression was used as a control for equal loading and to normalize the samples to one another.

### Immunofluorescent microscopy

The subcellular localization of transcription factors was visualized utilizing immunofluorescent microscopy with modification for cell type and instrumentation for STAT1, T-bet, STAT6, GATA3 and nucleus. Briefly, T cells were plated at standard concentration for 48 hours with designated experimental conditions. Cells were then lifted, washed twice with blocking buffer (BB), phosphate-buffered saline (PBS) with 1% goat serum (Hyclone, Logan, UT) and 1% horse serum (Hyclone), and then fixed with cold (−20°C) MeOH for 5 min. Cells were washed again with BB and then incubated for 40 min with 1:50 primary antibody at 4°C. STAT1, and 6, T-bet and GATA3 primary antibodies were obtained from Santa Cruz Biotechnology (Santa Cruz, CA). Cells were washed with BB, then incubated for 30 min with 1:50 fluorescent secondary antibody at 4°C protected from light. Cells were washed again with BB, resuspended in a minimal amount of Vectashield with DAPI (Vector Labs, Burlingame, CA), and then mounted in 35-mm culture plates (MatTek, Ashland, MA) with incorporated cover slip for microscopic imaging and kept at 4°C until imaging. The plates were imaged with a Zeiss LSM 510 NLO laser scanning confocal/multiphoton microscope (Zeiss, Thornwood, NY) equipped with a Coherent Chameleon tunable femtosecond Ti-Sapphire laser (Coherent, Santa Clara, CA) with META spectral detection (Zeiss). The various fluorescent channels were imaged simultaneously and digitally overlaid. We compared four treatment groups to see the effect of calpeptin on MBP specific cells and Th transcription factors. Unstimulated: no MBP-peptide, Stimulated: with MBP-peptide Stimulated+10 µM Calpeptin and Stimulated+1 µM Calpeptin.

### Statistical analysis

Data from various experiments were analyzed using SPSS software (SPSS, Chicago, IL) and shown as mean ± standard error of mean (SEM) of independent experiments (n ≥ 3). Statistical significance was determined by using either the unpaired Student’s t test or one way analysis of variance (ANOVA) with Games- Howell post hoc test at 95% confidence interval when multiple group comparisons were required. Statistical significance was obtained when p value was <0.05.

## Results

### Calpain inhibition attenuated inflammatory T cell proliferation and increased anti-inflammatory T cell types

**Figure 1:**
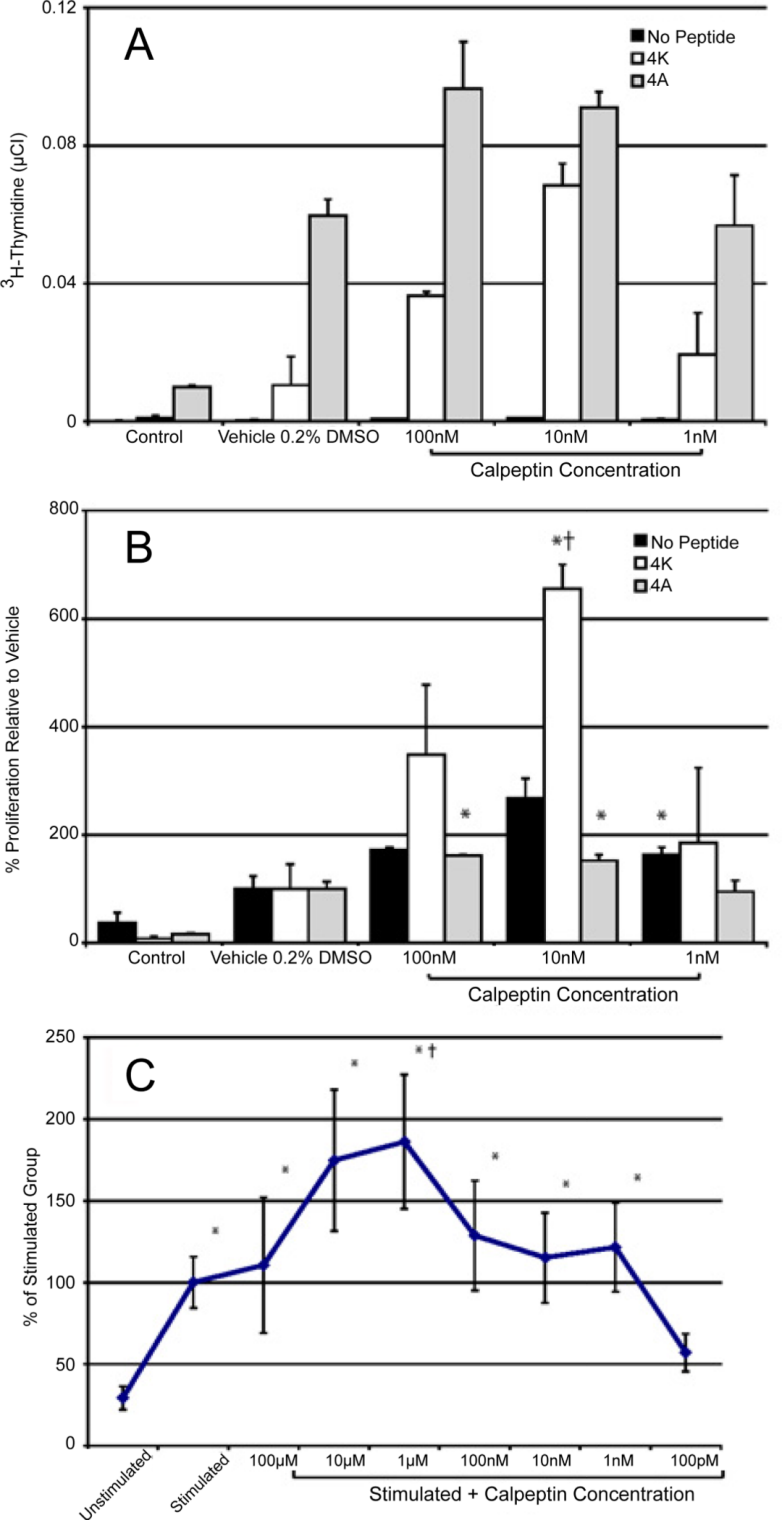
Calpain concentration dependent T cell proliferation Proliferation of MBP Ac1-11-specific T cells either in the presence of no antigenic peptide, the native MBP Ac1-11 (4K) peptide, or a strong agonist APL MBP Ac1-11 with an alanine substitution at position 4 (4A). Cells were incubated in the presence of several concentration of the calpain inhibitor calpeptin. **A.** The raw ^3^H-thymidine concentration. **B.** % ^3^H-Thymidine relative to the vehicle group set at 100. Mean ± SEM (n=3). *, p<0.05 vs. control; †, p<0.05 vs. vehicle group. **C.** MBP Ac1-11-specific cells were incubated in the presence or absence of the native MBP peptide (4K) and shown over several doses of calpeptin. Mean ± SEM (n = 4). *, p<0.05 vs. unstimulated control; †, p<0.05 vs. stimulated group.

We have previously shown that calpain is differentially involved in proliferation of human PBMCs and production of some T cell specific cytokines [31]. Based on these observations, we now examined the effect of calpain inhibition on proliferation of MBP-specific T cells (Figure 1). The proliferation of MBP Ac1-11-specific T cell line with various concentrations of agonists was measured to demonstrate the proliferation potential related to antigenic stimulation. Two peptides were chosen, altered peptide ligand (APL) 4A and MBP Ac1-11 with alanine at position 4, which binds to MHC II proteins much more strongly than the native MBP Ac1-11 peptide (4K). Proliferation of MBP-specific T cells treated with various concentrations of the calpain inhibitor calpeptin was tested and a concentration dependent proliferative response was observed (Figures 1A and 1B). The native 4K MBP peptide showed superior proliferation than the strong agonist 4A. Maximum proliferation of the cells stimulated with 4K was observed in presence of 10 nM calpeptin in the culture (Figure 1B).

The native 4K MBP peptide was chosen for further studies due to the graded effect. Proliferation experiments were then performed with a wider range of concentrations of calpeptin to determine if the proliferative effect was concentration dependent (Figure 1C). The proliferation of all of the groups except the 100 pM calpeptin group were significantly greater than the unstimulated group (Figure 1C). The groups trended toward increased proliferation at the midrange of calpeptin concentrations tested and returned to the calpeptin independent level of stimulation at the lower concentrations. The proliferation was significantly higher than the stimulated group when 1 µM calpeptin was also present in the culture. We further hypothesized based on calpain’s known involvement with STAT 6 that subtype of the proliferated cells would be Th2 [30,37].

**Figure 2:**
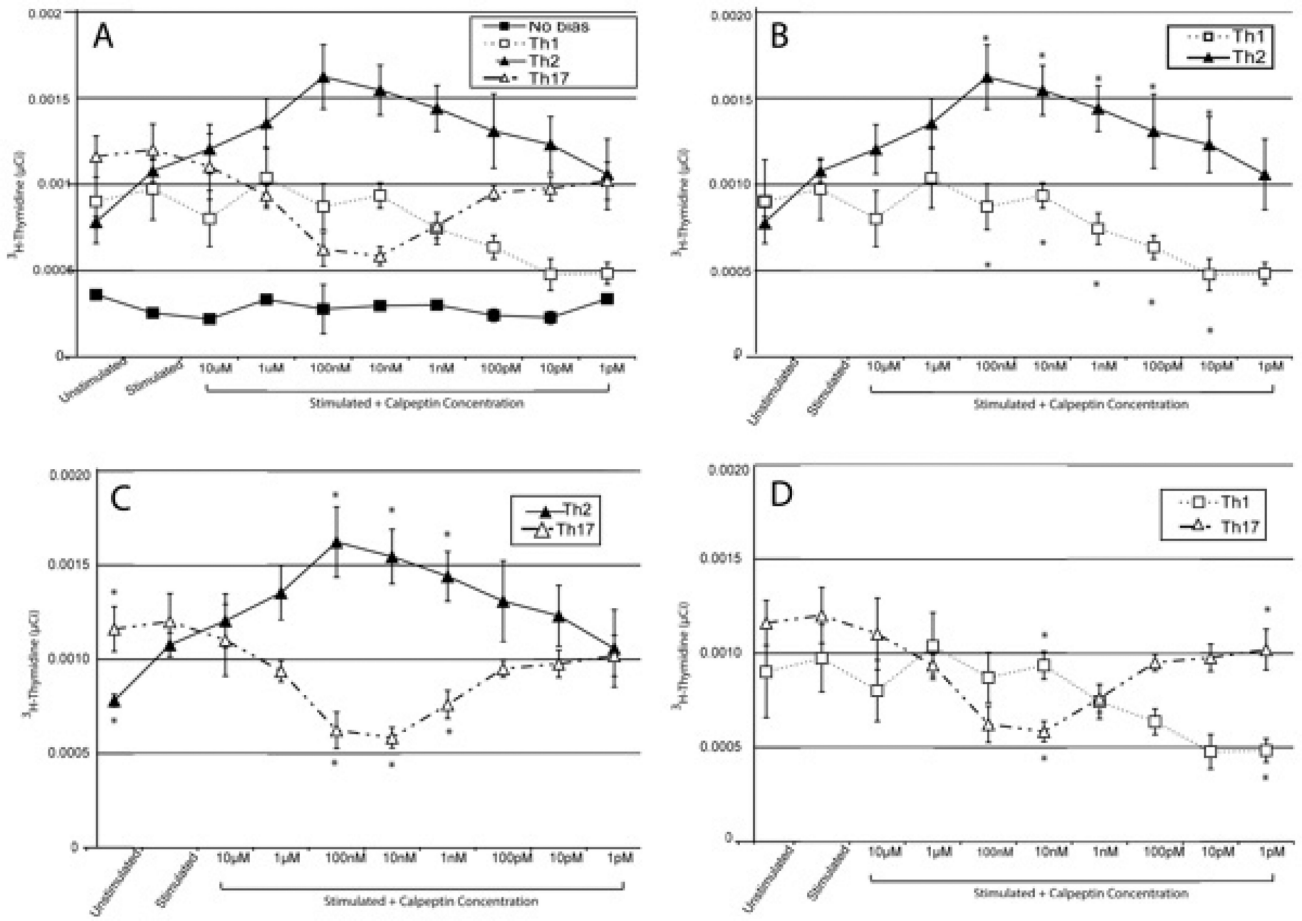
Th Subtype proliferation with calpeptin compared to each other A comparison of the proliferation of all the subtypes of Th cells responsive to MBP Ac1-8 as measured by ^3^H-thymidine uptake when stimulated with MBP Ac1-8 peptide in presence of various concentrations of the calpain inhibitor calpeptin. **A**. The various Th subtypes were potentiated by cytokines and antibodies in the media. Mean ± SEM (n=3). **B.** Comparison of the proliferation of Th1 biased cells with Th2 biased cells at each data point. **C.** Comparison of the proliferation of Th2 biased cells with Th17 biased cells at each data point. D. Comparison of the proliferation of Th1 biased cells with Th17 biased cells at each data point. Mean ± SEM (n=3). *, p<0.05 when compared between groups at the same treatment point.

In order to determine if the proliferative effect due to calpain inhibition was restricted to a particular Th subtype, we repeated the proliferation experiments on the various subtypes of Th cells. For this, we used primary cells that were isolated from draining LN and spleen from mice following MBP inoculation, and we then stimulated and polarized the cells *ex vivo*. When the proliferation of each subset was overlayed, a trend of increasing Th2 and decreasing Th1/Th17 cells was seen with given a calpain inhibition treatment (Figure 2A). When the proliferation experiment was performed without biasing cytokines or antibodies present in the media, there was no significant effect on proliferation from the various concentrations of calpeptin when compared with unstimulated or stimulated controls (Figure 2A). When the cells were cultured in the presence of the cytokines favoring the proliferation of the Th1 or Th17 (Figures 2B and 2D) subtypes, the addition of calpeptin either did not affect proliferation or decreased proliferation in comparison with the control cells. When the cells were cultured to favor Th2 subtype, the addition of calpeptin increased proliferation at the midrange of the inhibitor concentration (Figure 2C). The proliferation profile showed that the addition of calpeptin resulted in significantly increase in proliferation of Th2 cells in a concentration dependent manner while decreasing the inflammatory counterparts Th1 and Th17 cells.

### Calpain inhibition changes Th cytokine output

**Figure 3:**
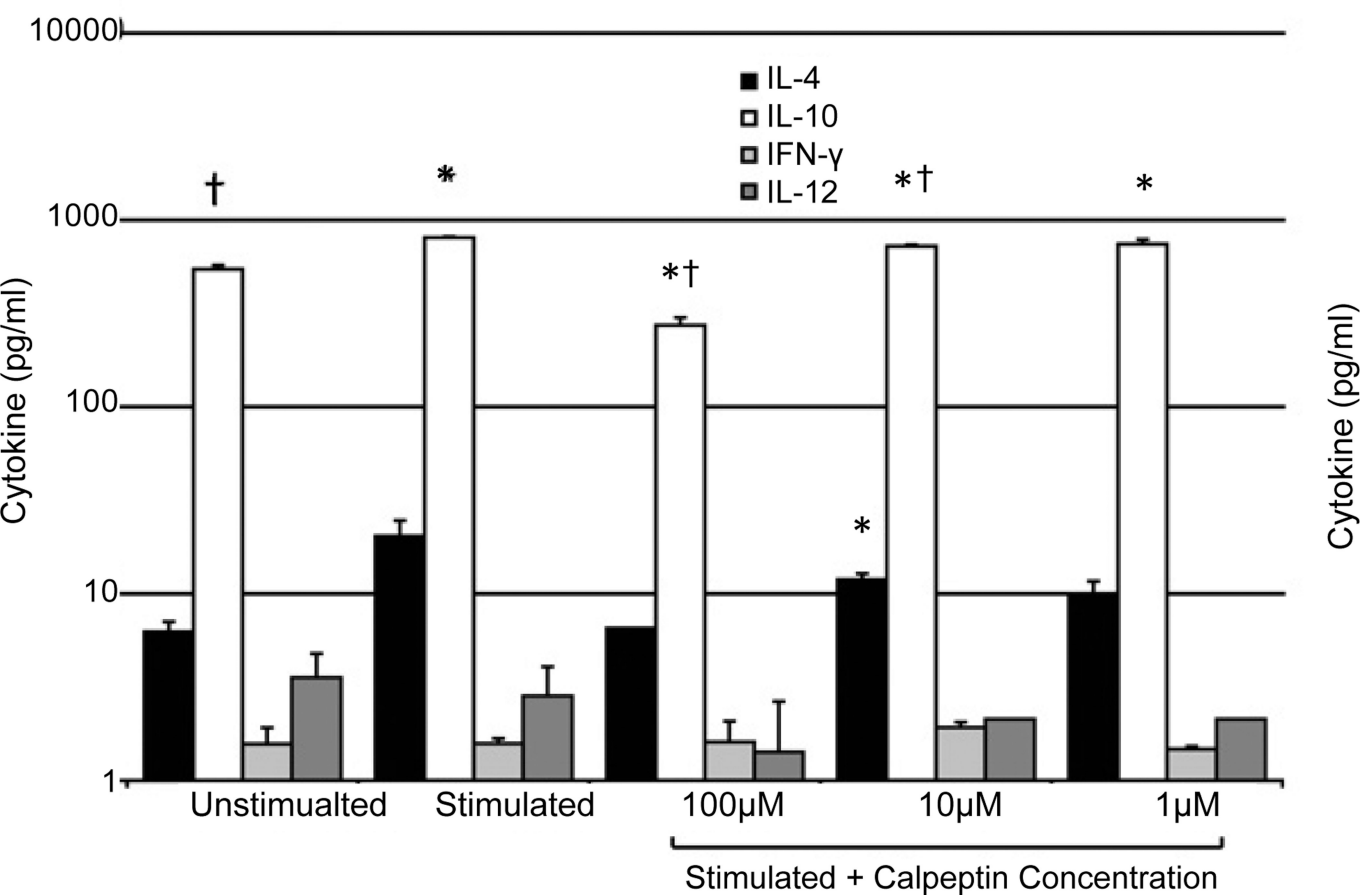
Calpeptin alters T cell cytokines **A.** Cytokines produced by MBP Ac1-11-specific T cells when stimulated with the native MBP Ac1-11 (4K) peptide in the presence of several concentrations of the calpain inhibitor calpeptin. Mean ± SEM (n=3). **B.** Cytokines produced by MBP Ac1-11-specific T cells when stimulated with the native MBP Ac1-11 (4K) peptide in the presence of various concentrations of calpeptin. Mean ± SEM (n=4). *, p<0.05 stimulated vs. unstimulated control; †, p<0.05 stimulated vs. stimulated control without calpeptin.

To further investigatethe Th2subtype after calpain inhibition, we examined key cytokine expression (Figure 3). The supernatants from stimulated cells treated or untreated with several concentrations of calpeptin were examined. Significant differences comparing the unstimulated and stimulated controls to the calpeptin groups were observed in the Th2 cytokines IL-4 and IL-10 (Figure 3). The IL-10 levels were significantly different from the unstimulated control in all treatment groups (Figure 3). The IL-10 level was significantly lower in the 100 µM and 10 µM calpeptin groups when compared with the stimulated group with no calpeptin. This was different than expected, which may be able to be explained by it being a co culture of Th subsets rather than the subsets being represented individually. In this same scenario, we saw Th1 cytokines, IFN-γ and IL-12, were extremely low in all the groups tested and no significant difference between two groups was observed.

**Figure 4:**
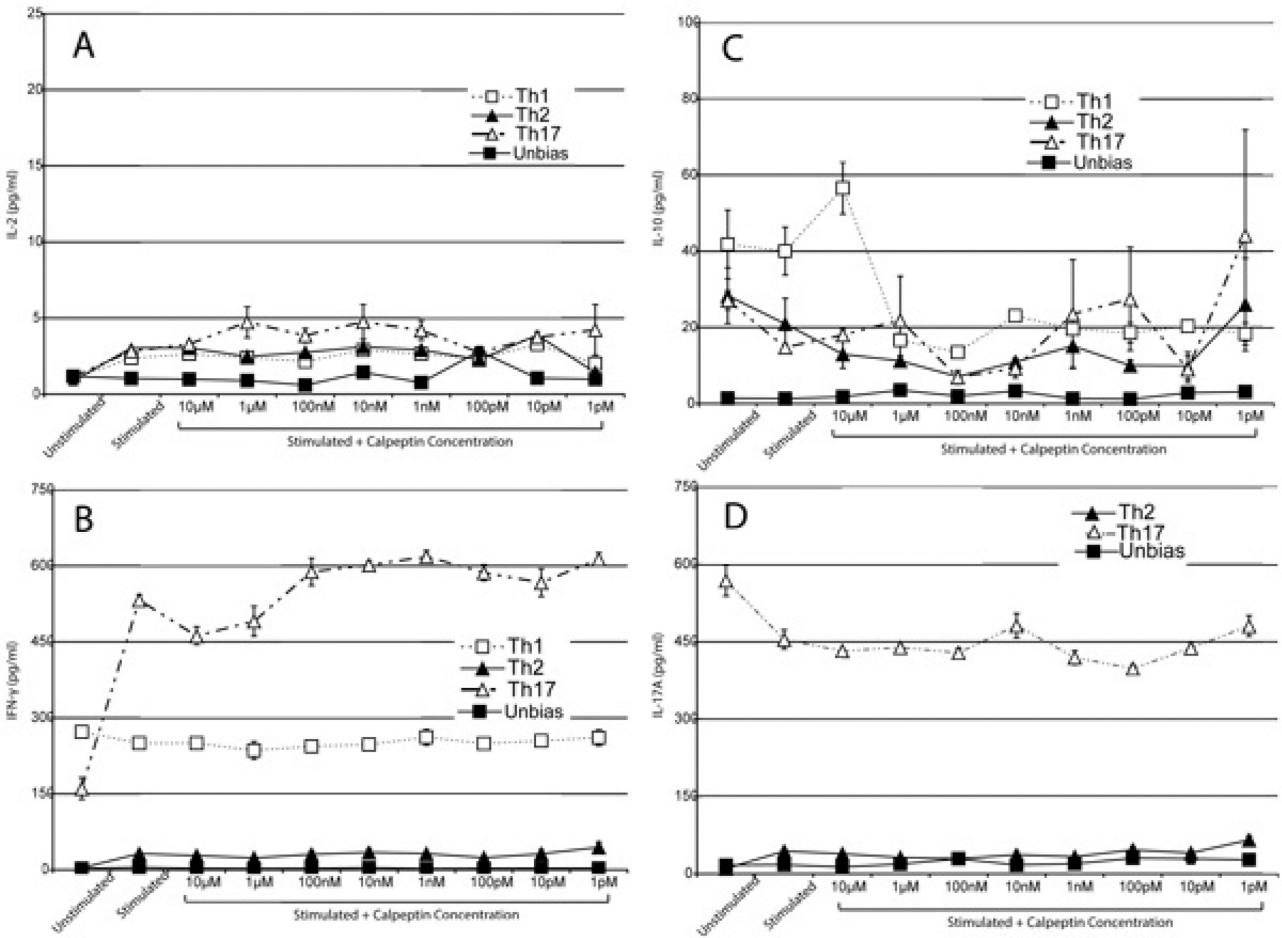
Th Subtype cytokines **A.** comparison of the cytokine production of all the subtypes of Th cells as measured by ELISA assay when stimulated with MBP Ac1-8 peptide in the presence of various concentrations of the calpain inhibitor calpeptin. The cytokine profiling was performed to determine if calpain inhibitor at various concentrations was having any effect on the cytokine production. A. compares the production of IL-2 for the various subtypes. **B.** compares the production of IFN-γ for the various subtypes. **C.** Comparison of the production of IL-10 in the various subtypes. **D.** Comparison of the production of IL-17A in the various subtypes. Mean ± SEM (n=3).

To study further, we polarized the cells and separated them into their own Th subsets. We then monitored the cytokine profiles of the primary MBP-sensitized biased cells. In these assays, we determinedIL-2, IFN-γ, IL-10, and IL-17A levels post polarization and treatment with decreasing calpeptin concentrations (Figure 4). While cytokine profiles were varied and hard to pin down, our data showed a trend towards an increase in anti-inflammatory cytokines during the calpain inhibitor treatment. IL-17 polarized cells showed a trend towards an increase in IL-2 production with calpeptin treatments of 10 µM, 1 µM, 100 nM, 10 nM and 1 nM calpeptin treatments compared to both the unstimulated and stimulated controls (Figure 4A). IFN-γ levels showed a strong increase from unstimulated control to stimulated control in Th17 polarized cells, however, post treatment with 10 µM and 1 µM, there was a reduction of IFN-γ. IL-17a levels in the Th17 polarized cells were also slightly reduced with calpeptin treatment at concentrations 10 µM, 1 µM and 100 nM as compared to stimulated controls. Taken together, some of the more concentrated calpeptin doses show a reduction of inflammatory cytokines, IFN-γ and IL-17a, and an increase in IL-2 production showing a trend toward anti-inflammatory mechanism of action.

### Calpain inhibition increased STAT6protein levels

**Figure 5:**
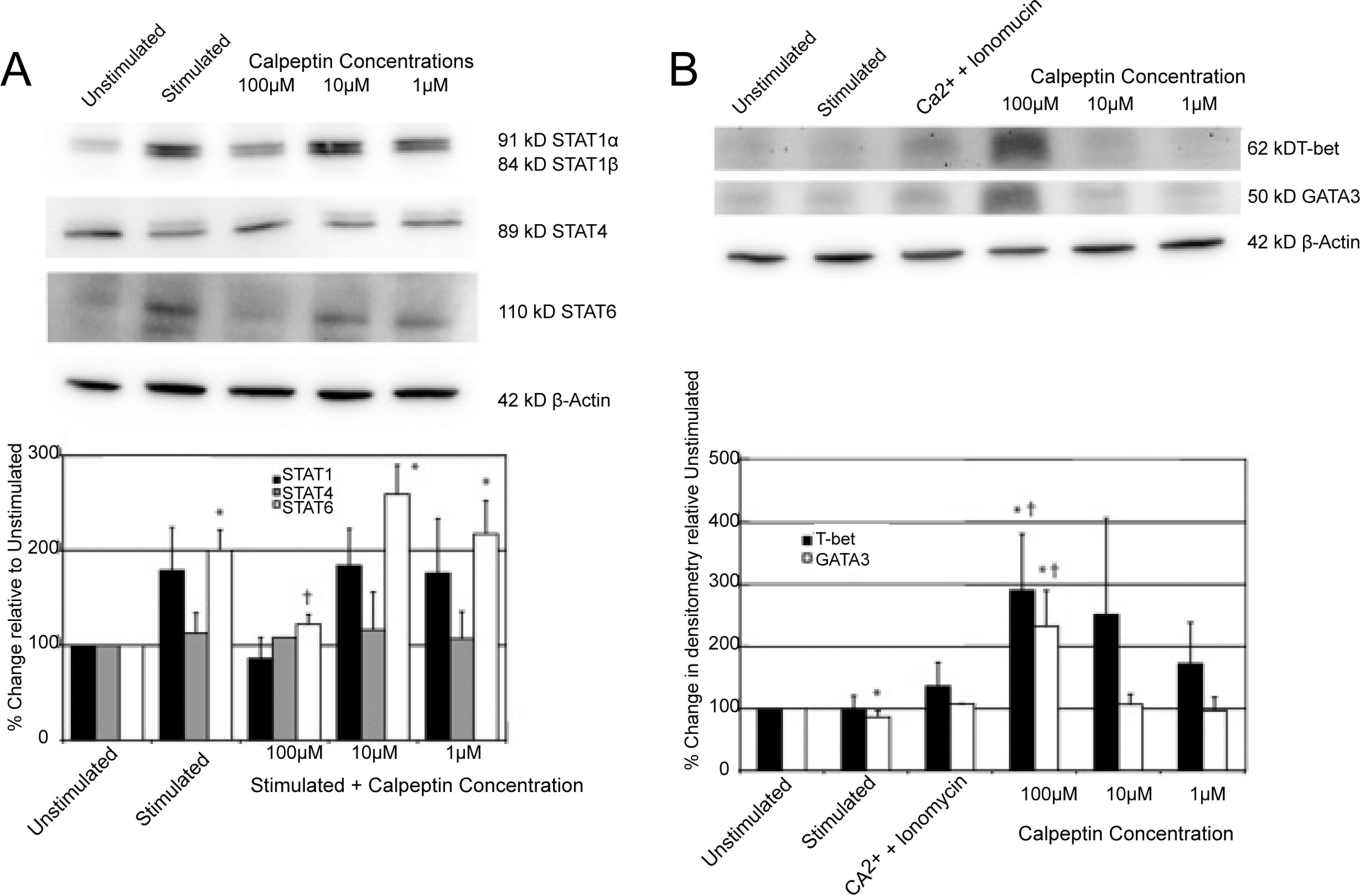
The effect of calpeptin on STAT protein levels Changes in various STAT proteins in MBP Ac1-11-specific T cells following stimulation with MBP Ac1-8 and various concentrations of the calpain inhibitor calpeptin. **A.** Representative Western blots showing 91kD STAT1α, 84 kD STAT1β, 89 kD STAT4, 110 kD STAT6 and 42 kD β-actin. Densitometric analysis showing percent changes in the various STAT proteins relative to the unstimulated control group set at 100. Mean ± SEM (n=4). *, p<0.05 stimulated vs. unstimulated control; †, p<0.05 stimulated vs. stimulated group without calpeptin. **B.** Changes in the T-bet and GATA3 transcription factors in MBP Ac1-11-specific T cells following stimulation with MBP Ac1-8 and various concentrations of the calpain inhibitor calpeptin. The Ca^2+^ + ionomycin group is a positive control group to contrast calpain activation to calpain inhibition. Representative Western blots showing 62 kD T-bet, 50 kD GATA3 and 42 kD β-actin. Densitometric analysis showing percent changes in the T-bet and GATA3 proteins relative to the unstimulated control group set at 100. Mean ± SEM (n=3). *, p<0.05 stimulated vs.unstimulated control; †, p<0.05 stimulated vs. stimulated group without calpeptin.

Calpain does appear to play a role in the Th cell bias but exactly where in the activation cascade is still unclear. In order to further determine where calpain may be playing a role in this bias, we examined the status of the numerous STAT signaling molecules. These STAT molecules, which are known to play a role in the Th cell subtype activation and proliferation by activating specific transcription factors, potentiate the different subtypes [38]. Whole cell lysates of MBP specific cells following exposure to activating stimuli and several concentrations of the calpain inhibitor were examined for STAT proteins (Figure 5). We specifically have looked at STAT1 and STAT4 that are important for Th1 subtype activation and STAT6 that is more important for Th2 subtype activation. There is no significant difference observed between the STAT1 and STAT4 protein levels for any of the groups tested, which is expected as they are not direct substrates of calpain (Figure 5A). STAT6, on the other hand, is a substrate of calpain. Interestingly, STAT6 expression was significantly increased when treated with 10 µM and 1 µM of calpeptin as compared with the unstimulated control group (Figure 5A). However, STAT6 protein level from the 100 µM calpeptin group was significantly lower than the stimulated control group. This may be due to the possibilities that the high level of calpain inhibitor affects a cell cycle mechanism and arrests a majority of the cells in a phase with low levels of STAT6 protein.

Looking further, we assessed certain transcription factors that are activated by STAT molecules and transcribe the cytokine loci necessary for the ongoing proliferation of the various subtypes. The main transcription factors that were measured in the MBP-specific cells were the Th1 and Th2 transcription factors T-bet and GATA3, respectively (Figure 5B). The calpain stimulated group with Ca^2+^ and ionomycin and the stimulated groups with 10 µM and 1 µM calpeptin were not significantly different than either of the unstimulated control or the stimulated group. When treated with 100 µM calpeptin, GATA3 was significantly increased (Figure 5B). For the same treatment group, T-bet was also significantly increased (Figure 5B).

From our data, we found that calpain inhibition at certain levels resulted in significant increases in STAT6, which could lead to Th2 increases. We also saw a significant increase in GATA3, a Th2 transcription factor. Although the data did not appear clear cut, we could see a strong trend toward increases in Th2 factors and decreases in Th1 with calpain inhibitor treatment.

### Effect of calpain inhibition on localization of transcription factor

**Figure 6:**
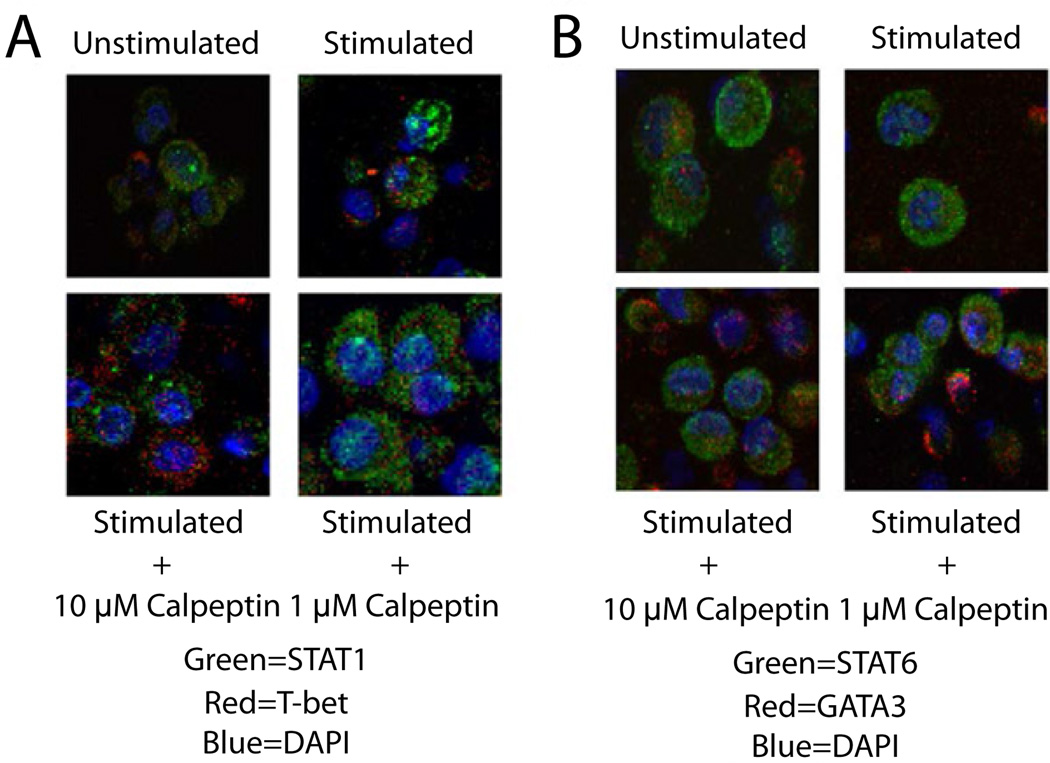
Immunofluorescent staining for localization of transcription factor Representative images of MBP Ac1-11-specific T cells stimulated with MBP Ac1-8 peptide for 48 hours and then stained with immunofluorescent staining for: **A.** STAT1 and T-bet and **B.** STAT6 and GATA3. The nucleus of the T cells was stained with DAPI. In both staining paradigms the calpeptin treated groups demonstrated a larger percentage of cells staining positive for the transcription factors T-bet and GATA3 when compared with untreated control and untreated stimulated group (n=3 for all treatments and staining groups).

We further investigated the proliferative effect observed in Th2 cells by calpain inhibition. The signal for proliferation and activation comes from surface receptors on the T cells and must travel to the nucleus in order to activate the necessary transcription factors. Fluorescent immunohistochemical staining was used to assess the subcellular localization of STAT1, STAT6, T-bet and GATA3 following stimulation with and without calpeptin. MBP Ac1-11 specific T cells stimulated with MBP Ac1-8 peptide for 48 hours showed increased expression of STAT-1 and T-bet, whereas calpeptin (1 µM) treatment reduced T-bet expression and cellular localization (Figure 6A). T cells stimulated with MBP Ac1-8 peptide also showed regular STAT-6 with no visible GATA-3 staining, but calpeptin treatment upregulated both STAT-6 and GATA-3 expression (Figure 6B). Although the control and treatment groups appear to show similar staining patterns, GATA3 expression seems to either surround the nucleus or reside in the periphery of the nucleus. These data suggest that calpain inhibition supports STAT-6/GATA-3-mediated Th2-transcription and proliferation, while downregulating T-bet-mediated Th1 transcription and proliferation.

## Discussion

MS is a multifaceted disease, involving interactions between the immune system and the CNS leading to axonal and neuronal degeneration caused by pro-inflammatory attack on the CNS. It is clear that MS has two arms to the disease, the activation, migration, and production of mediators by autoreactive T cells and other immune cells as the “immune arm” and axonal damage, demyelination, and neuronal and glial cell death as the “neurodegenerative arm”. The current therapies for MS primarily focus on attenuating the pro-inflammatory immunologic arm of the disease, but do not block relapse or progression of the disease. This treatment course allows neurodegeneration to continue and leads to disability in patients despite gold standard treatments. Therefore, new therapeutic strategies must be developed to block both inflammatory and neurodegenerative components of MS, which may help reduce or block disease progression and improve function. We have previously shown that calpain is involved in a multitude of cellular processes in many cell types [39–41]. Not only is calpain upregulated in the CNS of EAE animals with severe disease, but inhibiting calpain reduces the disease and preserves myelin. With neuroprotection in mind to help with the neurodegenerative arm of the disease, we sought in this study to look solely at the immune arm and mechanism through which calpain was involved in MS propogation.

The immune arm of the disease is largely mediated by helper T (Th) cells. Activation of naive CD4^+^ Th cell receptors, and co-receptors, leads to differential STAT signaling that drives lineage development through selective cytokine production. STAT 1 and 4 drive the development of inflammatory Th1 cells development while STAT3 drives development of inflammatory Th17 cells. Anti-inflammatory Th2 cells depend on STAT6 for development. It is believed that a shift from inflammatory associated STAT proteins (STATs 1,4,3) to non-inflammatory associated STAT proteins (STAT6) is correlated with a reduction of disease in EAE [30]. Along with STAT proteins, other transcription factors are highly associated with the development, commitment and survival of Th subtypes. While T-bet and ROR-γt/ RORC are associated with Th1 and Th17 inflammatory cells respectively, GATA3 and Foxp3 are non-inflammatory associated transcription factors for Th2 and Treg cells, respectively. Low levels of ROR-γt/ RORC and T-bet, and higher levels of STAT6, are associated with lower disease states in EAE. It is therefore inferred that decreasing the Th1 and Th17 inflammatory cells while increasing the Th2 and Treg cells can lead to decreased disease severity in EAE. STAT 6 is a known substrate of calpain. Previous studies have shown that activated STAT6 is degraded in T cell hybridoma cells and this degradation can be prevented with calpain inhibitors [28].

To look into the role calpain was playing in this tangled web of Th cell propagation, we investigated calpain inhibition outcomes on Th subsets. Our current data demonstrate that calpain plays an important role in Th subtype bias and potentiation of the Th2 subtype. The CD4^+^ MBP Ac1-11 T cell line demonstrated an increased proliferation when several concentrations of the calpain inhibitor calpeptin were included in the assay. This proliferative potential was observed both in the presence and absence of stimulation. The effect was not significant when the cells were not stimulated with their antigen but the trend was apparent. The effect of calpain inhibition on proliferation was more pronounced and significant during stimulation providing evidence that calpain signaling might act in the activation pathway of this Th subtype. This experiment with different methods for activating the MBP Ac1-11-specific T cells also demonstrated that over activation could drown out the proliferative effect seen with the calpain inhibitor. The distinctly different proliferative profiles observed in the Th subtypes with various calpeptin concentration ssupports the evidence that calpain plays a role in Th2 cell subtype as well as in favor of the evidence that the MBP Ac1-11-specific cell line is of a Th2 subtype lineage.

Previous research in our laboratory on calpain inhibitor in the EAE model of MS has demonstrated a reduction of clinical signs and disease severity with treatment [33]. The clinical signs and relapse were reduced in an adoptive transfer model of EAE in which the primed T cells were exposed to calpain inhibitor before transfer to the susceptible animals [33]. Calpain appears to act as a negative regulator of Th2 cells through the cleavage of STAT6. Calpain is also known to be upregulated in activated Th1 cells before the onset of clinical disease [42]. Therefore, the balance of calpain activity probably plays a role in Th cell bias in response to immune challenge contributing to the proper immune response.

Recently, the importance of Th17 cells in the pathogenesis of EAE has been demonstrated and the decrease in Th17 subtype proliferation is another potential mechanism by which calpain inhibitor decreases pathogenesis of EAE. The primary cell subtypes were compared with each other in order to demonstrate that the significance of calpain inhibition on proliferation varied by subtype. The proliferation is important for the cell to become the dominate subtype but cytokines also play a role in the inflammatory environment during an immune reaction, so they are also explored in the context of calpain inhibition. There was no significant difference observed with IL-4 and the only significant difference observed with IL-10 was with the group exposed to 10 nM calpeptin. This cytokine observation was repeated in the primary cell cultures in which the cytokines were measured. We have found that the subtypes are in fact becoming the dominant subtype in their respective polarized cultures by the profile measured. However, calpain inhibitor treatment did not produce any significant alteration at most of the concentrations tested. Several of the subtype cytokines that would have been interesting to test were tested in the cells; IL-4 could not be tested in the primary culture because exogenous cytokines were added as part of the assay. While these assays reveal that calpain is involved in Th cell bias, they do not shed significant light on the possible mechanism, suggesting that careful examination of primary cells with limited exogenous factors are needed. Our study, however, demonstrate that cytokine secretion is preserved. The preservation of cytokine secretion at stimulation levels is important in order to maintain proper T cell function and the proper inflammatory environment. The cytokine profile is reinforced by the cytokines binding to receptors that activate STAT molecules, which in turn transcribe cytokine loci to further strengthen a hypothesis of polarization and subtype generation of T cells by calpain inhibition.

Calpain is also known to act upon several of these STAT molecules so the proliferative and cytokine effects observed in these MBP-specific T cells by inhibiting calpain activity may be related. The levels of STAT proteins in the whole cell lysate were examined following activation in the presence of different concentrations of calpeptin. The effect of reduced cytokines observed at the highest concentration of calpeptin is repeated with the STAT protein levels at the highest concentration of calpeptin to compare with the stimulated control. The STAT1 level and STAT4 trend were lower but the level of STAT6 was significantly lower when compared with the stimulated control. This reduction in STAT protein levels at the highest concentration of calpeptin is probably due to non-specific cell cycle effects and the arrest of the cells in a phase in which the STAT proteins exist at a low level. The two lower concentrations of calpeptin (10 µM and 1 µM), however, result in STAT6 levels that trend higher than the stimulated control and are significantly higher than the unstimulated control. This is in contrast to the highest calpeptin concentration. This result further supports the hypothesis that calpain contributes to subtype bias through STAT6. This also correlates with the proliferation assay where proliferation of the E3 cells is increased with 10 µM and 1 µM calpeptin.

If calpain is acting to repress the Th2 subtype through cleavage of STAT6 and calpain inhibition results in increased proliferation of a Th2 subtype as well as increased STAT6 protein levels, it follows that the transcription factors downstream of STAT6 may be increased as well. This notion was explored by looking at levels of transcription factors T-bet (Th1) and GATA3 (Th2) following stimulation in the presence of various concentrations of the calpain inhibitor. There was a significant increase of T-bet and GATA3 protein levels in the groups exposed to the highest calpeptin concentration (100 µM) when compared with the unstimulated and stimulated controls. The reason why T-bet follows a somewhat similar trend to GATA3 can be ascribed to the idea that the protein levels may be controlled in some way by the activity of proteases in the cells. The activity and involvement of the protein to carry out its role of transcription and increasing certain cytokine concentrations may lie with a different signaling mechanism.

The subcellular localization of the STAT proteins and transcription factors was determined to discern if the location of these proteins in the cells played a more important role than just the quantity of the protein themselves. We revealed the increased staining of both transcription factors in the groups that were incubated with calpeptin, especially in the case of GATA3. The transcription factor staining also appeared more perinuclear in the stimulated cell groups when compared with the unstimulated control group. This fits with the mechanism that a transcription factor may move to the nucleus in order to accomplish its task when the cell becomes activated. The STAT proteins also display a more punctate staining pattern in the stimulated groups when compared with the unstimulated control which stains more diffuse through the cytoplasm. This pattern would correspond to increased concentrations of the protein at cytokine loci within the nucleus.

In conclusion, our current findings confirm the concept that calpain is involved with Th cell bias as we observed in 2 different cell systems. Calpain inhibition increases the Th2 subtype by increasing proliferation but not by increasing cytokine secretion. The mechanism by which this occurs may be due in part to the involvement of calpain in the STAT signaling pathway. Our data also show that STAT6 with slight increases in concentration is localized in the nucleus, suggesting that localization over concentration of this transcription factor is the key feature to create and maintain the Th subsets. Calpain is indeed involved in this tangled web of Th inflammatory processes and could be a potential double barrel target to influence both the immune and neurodegenerative arms of MS disease.
